# Exploring the Role of Temporoparietal Cortex in Upright Perception and the Link With Torsional Eye Position

**DOI:** 10.3389/fneur.2018.00192

**Published:** 2018-04-06

**Authors:** Jorge Otero-Millan, Ariel Winnick, Amir Kheradmand

**Affiliations:** ^1^Department of Neurology, Johns Hopkins University School of Medicine, Baltimore, MD, United States; ^2^Department of Otolaryngology-Head and Neck Surgery, Johns Hopkins University School of Medicine, Baltimore, MD, United States

**Keywords:** ocular torsion, subjective visual vertical, temporoparietal cortex, transcranial magnetic stimulation, upright perception

## Abstract

Upright perception is a key aspect of orientation constancy, as we maintain a stable perception of the world despite continuous movements of our eyes, head, and body. Torsional position of the eyes can impact perception of upright by changing orientation of the images on the retina relative to gravity. Here, we investigated the role of temporoparietal cortex in upright perception with respect to ocular torsion, by means of the inhibitory effect of continuous theta burst transcranial magnetic stimulation (TMS). We used a subjective visual vertical (SVV) paradigm to track changes in upright perception, and a custom video method to track ocular torsion simultaneously. Twelve participants were tested during a lateral head tilt of 20° to the left. TMS at the posterior aspect of the supramarginal gyrus (SMGp) resulted in an average SVV shift in the opposite direction of the head tilt compared to a sham stimulation (1.8°). Ocular torsion following TMS at SMGp showed no significant change compared to the sham stimulation (−0.1°). Thus, changes in upright perception at SMGp were dissociated from ocular torsion. This finding suggests that perception of upright at SMGp is primarily related to sensory processing for spatial orientation, as opposed to subcortical regions that have direct influence on ocular torsion.

## Introduction

Human studies have shown a multisensory role of the temporoparietal cortex in perception of spatial orientation (1–6). There is mounting evidence that areas within the temporoparietal cortex such as the posterior insula, inferior parietal lobule (angular and supramarginal gyri), and superior temporal gyrus are involved in processing or encoding vestibular, visual and somatosensory information. However, the underlying mechanisms for convergence of these sensory information are poorly understood. Multisensory integration is indeed vital for orientation constancy since our eye, head and body positions change frequently. Such orientation constancy allows us to inherently perceive the world in upright orientation, despite the changing position of the images on the retina.

Perception of upright can be measured by a psychophysical task known as the subjective visual vertical (SVV). In this task, a visual line is used to measure the perceived earth-vertical orientation (7–10). When the head is tilted laterally toward the shoulder, the eyes roll in the opposite direction of the head tilt. This counter-rolling of the eyes does not fully compensate for the amount of head tilt (typically only by about 10–25%). Such partial compensation in torsional eye position results in deviation of the vertical meridian of the eyes, and subsequently tilt of the retinal images with respect to the axis of gravity. For example, if the head is tilted 20°, the eyes may rotate only 5°, and therefore the orientation of the retina relative to gravity would be 15°. Now in order to maintain accurate upright perception, the brain must determine the orientation of the images on the retina relative to gravity by integrating information from the head and body positions in space with the eye position in head. Thus, ocular torsion plays a critical role in perception of upright by affecting the orientation of the images on the retina.

Subjective visual vertical deviations from subcortical brain lesions are often accompanied by deviations in ocular torsion (11). Thereby, in such cases errors of upright perception are corollary to changes in the torsional eye position. Here we investigated a similar link between perception of upright and ocular torsion at the level of cerebral cortex, asking whether the cortical mechanisms involved in perception of upright directly affect ocular torsion, or instead these ‘higher-order’ mechanisms are primarily involved in processing sensory signals encoding visuospatial orientation. In order to address this question, we used an inhibitory effect of transcranial magnetic stimulation (TMS) at the posterior aspect of the right supramarginal gyrus (SMGp) within the temporoparietal junction (TPJ). We have previously shown that SMGp is involved in perception of upright orientation and likely has a role in maintaining orientation constancy (12). The inhibitory effect of TMS at this cortical location results in a shift of SVV errors in the opposite direction of the head tilt. Here, we used the same TMS method while recording SVV and ocular torsion simultaneously. A psychophysical paradigm was used for tracking changes in SVV responses, and the torsional position of the eyes was recorded using real-time video-oculography.

## Materials and Methods

### Participants

Twelve right-handed volunteers (mean age 26 years; nine females) participated in the experiment after giving written consent. All participants were in good health without vestibular, neurologic, or psychiatric illness. The inclusion criteria were based on the consensus guidelines for TMS use in research (13). All participants had normal ocular counter-roll in response to the 20° head tilts (4.6°± 0.6°) (14). The experiment procedures were approved by Johns Hopkins Institutional Review Board.

### General Experiment Procedures

**Figure 1:**
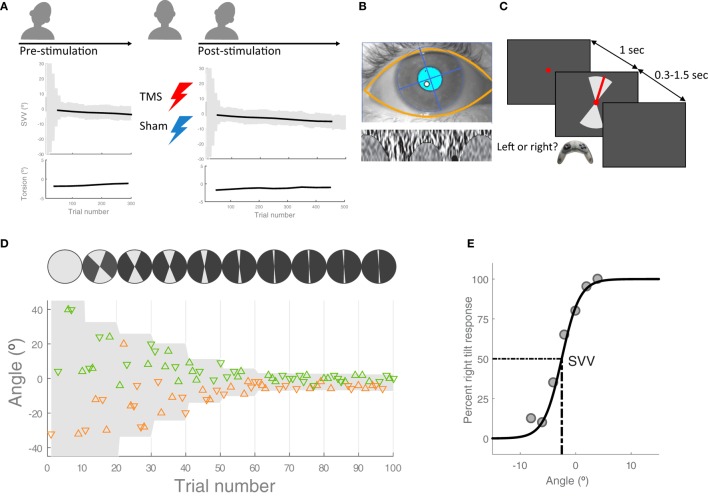
Experiment procedures: **(A)** in each session, first, the head was tilted 20° to the left and 300 subjective visual vertical (SVV) trials were recorded along with ocular torsion as the baseline values before transcranial magnetic stimulation (TMS) or sham stimulation [see **(C,D)** for details of SVV paradigm]. The head was then brought to upright position in order to apply TMS or sham stimulation. Following each stimulation, the head was tilted again and SVV and ocular torsion were recorded for 500 trials. These pre- and post-stimulation values were used to calculate changes in SVV and ocular torsion. **(B)** Ocular torsion was measured using iris tracking based on polar transformation of infrared images of the iris. An example eye image captured by this VOG method is shown, with the overlay of automatically detected eyelids and pupil (top) and the extracted iris pattern optimized to calculate ocular torsion (bottom). For more details, see Ref. (16). **(C)** SVV recording: in each trial, participants fixated on a red dot for 1 s before the line appeared. They had 1.5 s to respond if the line was tilted to the left or to the right of their perceived upright orientation (2-AFC). This was done by pressing the left or right button on a controller. After pressing the button, the line disappeared and the next trial started with a new line orientation. The line was presented within a range of angles (gray shade), which was adjusted every 10 trials. **(D)** SVV paradigm: a sample of responses for the first 100 trials is shown. The *y*-axis shows the line angles and each triangle represents one trial. The triangles that point up are the trials when the line was presented in the upper visual hemifield and the triangles that point down are the trials when the line was presented in the lower visual hemifield. The left tilt responses are in orange and the right tilt responses are in green. In order to track SVV changes, the line angles were presented randomly within a range that started at 360° and then adjusted in subsequent blocks (i.e., every 10 trials) based on the responses from previous blocks. **(E)** Example of SVV calculation: The center of a psychometric function fit was used to calculate SVV values in windows of 100 trials.

All participants underwent at least four separate experimental sessions, completing TMS and sham stimulation on different days (Figure 1A). One preliminary session without any stimulation was used to familiarize the participants with the psychophysical paradigm (data not shown in this study). Experiments were performed in a completely dark room while SVV and ocular torsion were measured simultaneously. In all sessions, the head was immobilized using a molded bite bar, which was mounted on a rotary motor (Zaber Technologies Inc., Vancouver, BC, Canada) so that the head tilt position could be controlled remotely. The head was tilted 20° to the left side, while participants were sitting upright. Tilting of the head was to induce changes in ocular torsion and also to lower the threshold for the effect of TMS on SVV errors, replicating the conditions of our previous study (12). In all sessions, we recorded SVV and ocular torsion for 300 trials before any stimulation was applied in order to obtain baseline, pre-stimulation values (see SVV paradigm and ocular torsion measurement below for details). After recording the baseline trials, the head was brought back to upright position and TMS or sham stimulation was applied while participants stayed on the bite bar in dim light during the stimulation. We used a continuous theta burst stimulation (cTBS) protocol, which transiently disrupts cortical activity with an inhibitory TMS effect lasting for minutes after the stimulation (12, 15) (see TMS and sham stimulation protocols below for details). Anatomical landmarks on the brain MRI were used to select the cortical locations for magnetic stimulation (see localization of SMGp below). Following TMS and sham stimulation, the bite bar returned to the 20° left tilt position and both SVV and ocular torsion were recorded for 500 trials in order to obtain post-stimulation values. The pre-stimulation SVV and ocular torsion values were then subtracted from the post-stimulation values to calculate respective shifts in SVV and ocular torsion following each stimulation (see data analysis below for details). Every time the head moved from upright to the tilt position, there was a 30-s pause in the SVV paradigm in order to avoid the residual effects of semicircular canal stimulations during head tilt on SVV responses.

### SVV Paradigm

We used a two-alternative forced choice paradigm (2-AFC) for SVV measurement in a completely dark room (Figures 1C,D) as reported in a previous study (10). The head was immobilized with a molded bite bar while the line stimulus was displayed on a CRT monitor (1,280 px × 1,024 px), 135 cm away in front of the participant. To eliminate all possible visual cues during the recordings, we set the brightness and contrast levels of the screen to minimum (screen luminance <0.5 cd/m^2^). The room had no windows and was specially designed to perform experiments in the dark with all walls, floor, and ceiling painted black and doors sealed using thick drapes. To further eliminate any potential cue coming from the monitor in the case of dark adaptation, we also used a black cardboard with a circular opening around the fixation spot. During each trial, the task was to click the right or left button on a controller to report whether the line was tilted to the right or left of perceived upright orientation. The line angle was randomly selected within a range of angles that was adjusted in blocks of 10 trials (Figure 1D). The fixation dot appeared first, and after 1 s, the line stimulus was presented for a minimum of 300 ms and maximum of 1.5 s until participants responded. If there was no response within 1.5 s, the line disappeared and a new trial started after clicking a button on the controller. In such cases, the missed angle (i.e., the line orientation) was presented again at a later time within the same block (i.e., 10 trials), ensuring that all the angles were presented and the corresponding responses were obtained exactly once. In each block, five different line angles were presented in the upper visual hemifield (always radiating from the fixation point), and the same five angles were presented in the lower visual hemifield. At the beginning of the paradigm (i.e., the first block), the angles were selected from the entire range of 360°, but this range was adjusted in subsequent blocks in order to track changes in upright perception. The adjustment was made by calculating a new range for each block, centered at the SVV value derived from the trial responses in previous three blocks (for SVV calculation see the data analysis). The width of the range decreased by half in every block until the paradigm reached the 9th block, after which it remained constant at 10° for all the remaining blocks. Thereby, the SVV paradigm could track changes in upright perception and it was not biased by making prior assumptions about the SVV value.

### Ocular Torsion Measurement

We used RealEyes xDVR system manufactured by Micromedical Technologies Inc., and a custom software to record torsional eye position. This video-oculography system uses two cameras (Firefly MV, PointGrey Research Inc., Richmond, BC, Canada) mounted on goggles to capture infrared images of each eye. Participants wore the goggles during the entire experiment sessions. In order to measure and track torsional eye position, we used a method based on iris recognition, which operates binocularly in real time at 100 Hz with a noise level that can reach less than 0.1° (16) (Figure 1B).

### TMS and Sham Stimulation Protocols

Each participant underwent a T1-weighted, high-resolution MRI using a 3T scanner (Philips Healthcare, Cleveland, OH, USA). We used a frameless neuronavigation system based on a 3D model of the brain (Brainsight, Rogue Research Inc., Montreal, QC, Canada) for real-time tracking of TMS coil and accurate targeting of a cortical area of interest. In this neuro-navigation method, a series of scalp landmarks on the brain MRI and the participant’s head are co-registered using infrared sensors. We used a Magstim Rapid^2^ stimulator and 70 mm figure-of-eight coil (Magstim Co., Whitland, UK) for TMS application. A train of 200 bursts was given at 5 Hz (inter-burst interval of 160 ms) for 40 s. Each burst consisted of three pulses repeating at 50 Hz for duration of 40 ms (total of 600 pulses). Participants wore ear plugs during TMS to dampen the noise from the coil discharges. The frequency, intensity, and duration of this cTBS protocol was within the safe limits, and there were no side effects from TMS in our participants (13).

To apply TMS, the coil was held tangential to the surface of the scalp by an articulated stand. The coil handle pointed backward, parallel to the Sylvian fissure for magnetic stimulation. The stimulation point (i.e., the center of the TMS coil) was continuously monitored using the neuronavigation system to ensure it remained directly over the cortical target location. The magnetic field generated by this method has an estimated spatial resolution of 1–2 cm^2^ with a depth of penetration about 2 cm below the scalp (17, 18). In each participant, we recorded active motor threshold (AMT), which is the lowest intensity of magnetic stimulation over the cortical hand area that can induce myogenic evoked potentials during isometric contraction of the first dorsal interosseous muscle. Although the intensity of magnetic stimulation is usually adjusted using AMT, it is not clear whether it would be relevant to the effects of TMS at sensory cortical regions (19, 20). Here, we used a fixed magnetic stimulation at 55% of the maximum stimulator output. The range of AMT was between 43 and 73% in our participants. Thus, the 55% fixed stimulation intensity corresponds to 75–127% of AMT across our participants. The procedures for sham stimulation was similar to the TMS except that a wooden block was placed between the TMS coil and the scalp, with the center of the coil oriented 90° away from the trajectory used for TMS application.

### Localization of SMGp

**Figure 2:**
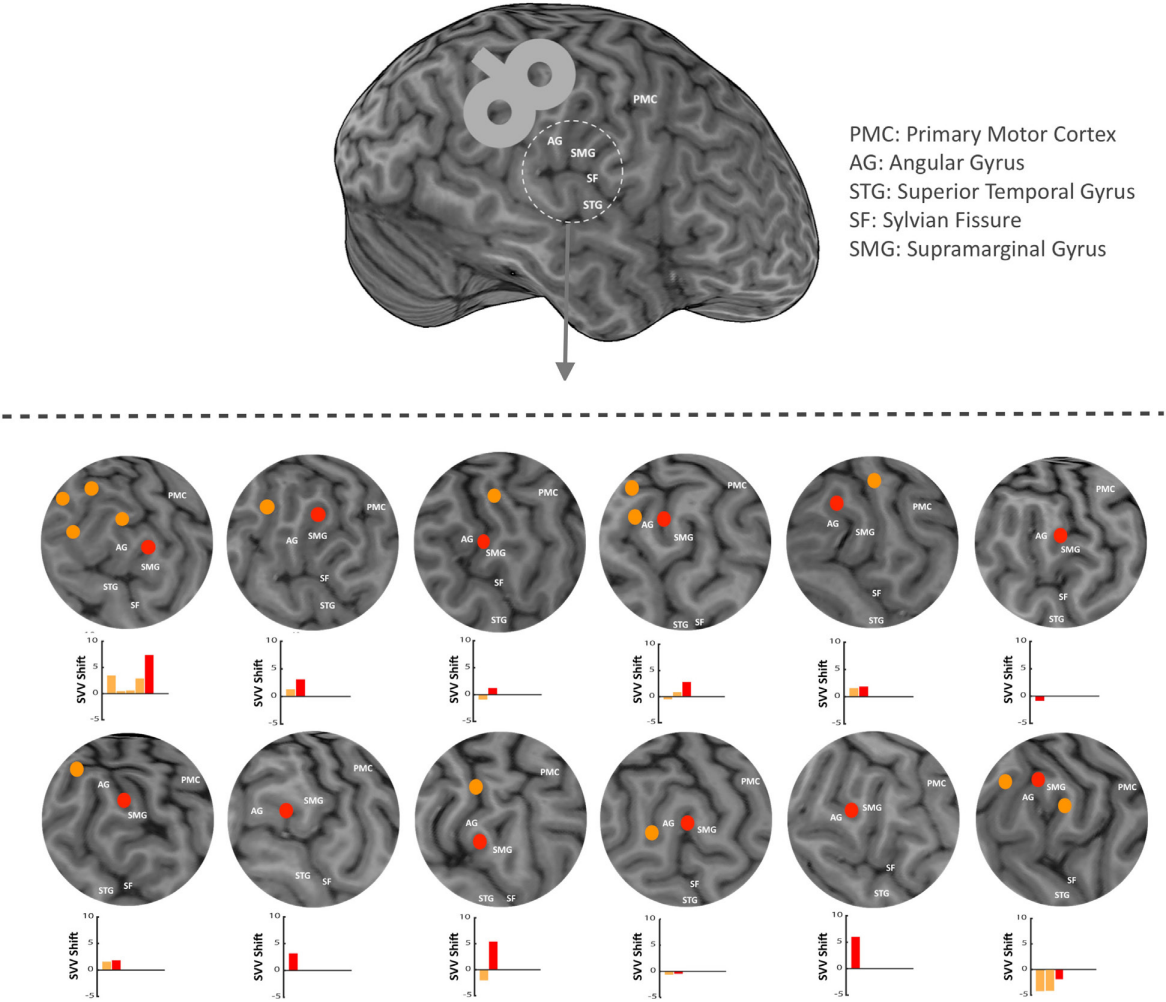
In each participant, anatomical landmarks were used on the brain MRI to estimate the location of the supramarginal gyrus (SMGp) within the right temporoparietal junction (TPJ). TMS was applied around the right supramarginal gyrus in order to functionally verify the location of SMGp through the effect of TMS on SVV responses (using real-time neuronavigation). Red dots show SMGp and orange dots show other probed locations within the TPJ. The corresponding SVV shifts are also shown for all locations. Here, the SVV shift is shown as the difference between the averages of post- and pre-TMS values. A positive SVV shift indicates a right shift and a negative SVV shift indicates a left shift. The SVV shift at SMGp (red bar) has a more positive value relative to the other cortical locations (orange bars), which means it is in the opposite direction of the head tilt (i.e., a right shift).

In each participant, anatomical landmarks were used on the brain MRI to estimate the location of the right SMGp. This was done by functionally identifying the right primary motor cortex and recording AMTs at the hand-knob area. The location of central sulcus could then be verified, and based on that, other cortical landmarks were identified including the supramarginal gyrus, angular gyrus, and posterior aspect of the supramarginal gyrus (SMGp) (Figure 2). Because the anatomy of the cortex varies greatly in this region among individuals, as a first step, we functionally verified the location of SMGp by applying TMS at the right SMGp and measuring its effect on SVV responses. As shown previously, the inhibitory effect of TMS at SMGp produces an SVV shift in the opposite direction of the head tilt (12). Here, we used a similar approach and measured the SVV shift following TMS in order to verify the location of SMGp. Figure 2 shows the cortical locations that were examined within the temporoparietal cortex along with the corresponding SVV shifts in each participant. On average, two cortical locations were examined among all participants (minimum one and maximum five locations). In five participants, the location of SMGp was verified after other cortical locations were examined, and in four participants, it was verified before other cortical locations were examined. Three participants had TMS only at SMGp and not at any other cortical location. The average MNI coordinates (Montreal Neurological Institute, 152 template) for the functionally verified SMGp location among all participants (red marks in Figure 2) were *X* = 55.4 (range 48.7–61.0), *Y* = −25.9 (range −34.0 to −12.8), and *Z* = 33.8 (range 24.6–47.0). Once we identified SMGp, a second TMS session was recorded at the same SMGp location in order to record SVV and ocular torsion simultaneously (as described in the experiment procedures). This second TMS session was done to ensure that the SVV shift at SMGp could be reproduced, as in some participants there was a selection bias toward a larger SVV shift in the first TMS session in order to identify SMGp from all the examined cortical locations.

### Data Analysis

SVV was calculated by fitting a psychometric curve to the forced-choice responses of every 100 trials, using a logistic function and a generalized linear regression model (Matlab fitglm). The SVV value was the angle at which the probability of left or right responses was 50% (point of subjective equality) (Figure 1E). In nearly all blocks, two or more angles had response rates between 11 and 89%, which confirms that the resolution of the probing blocks in the SVV paradigm was not low to cause biases by a few angles. We also examined SVV precision, which was calculated as the difference between the 50 and 75% points on the psychometric curve.

In order to compare the ocular torsion and SVV responses, we first calculated the average torsional position of both eyes during each trial in the SVV paradigm. These torsion values were then averaged within a sliding window of 100 trials. The corresponding SVV value was also derived from the psychometric curve within the same sliding window of 100 trials. This window of trials—in which SVV and torsion values were calculated—was then advanced in steps of 10 trials so that changes in SVV and ocular torsion could be displayed over time. The average pre-stimulation values were subtracted from the post-stimulation values to calculate the respective shifts in SVV and ocular torsion. These shift values were used for statistical comparisons. We used a paired *t*-test with a significance level of 0.05, as the SVV and torsion data were both normally distributed (Shapiro–Wilk *p* > 0.1). To compare changes in SVV and ocular torsion over time, we first fitted a linear regression and then compared the slopes using a paired *t*-test. In all sessions, SVV responses from the first 50 trials were discarded along with the ocular torsion recordings, as the ranges of probing angles in these initial trials were not narrow enough to obtain an accurate SVV value (see Figure 1D).

## Results

Here, the goal was to determine whether or not the shift in upright perception from the cortical effect of TMS at SMGp could be due to induced changes in ocular torsion. Accordingly, we used simultaneous SVV and ocular torsion measurements before and after applying magnetic and sham cortical stimulations. In each recording session, we accounted for baseline SVV and ocular torsion values to determine the effects of TMS and sham stimulations. Thus, SVV and ocular torsion shifts were calculated by subtracting the average pre-stimulation values from the post-stimulation values, which also eliminated the variable effect of head tilt across participants. In order to determine the specific effect of TMS, the corresponding shifts in SVV and ocular torsion were compared to the sham stimulation.

**Figure 3:**
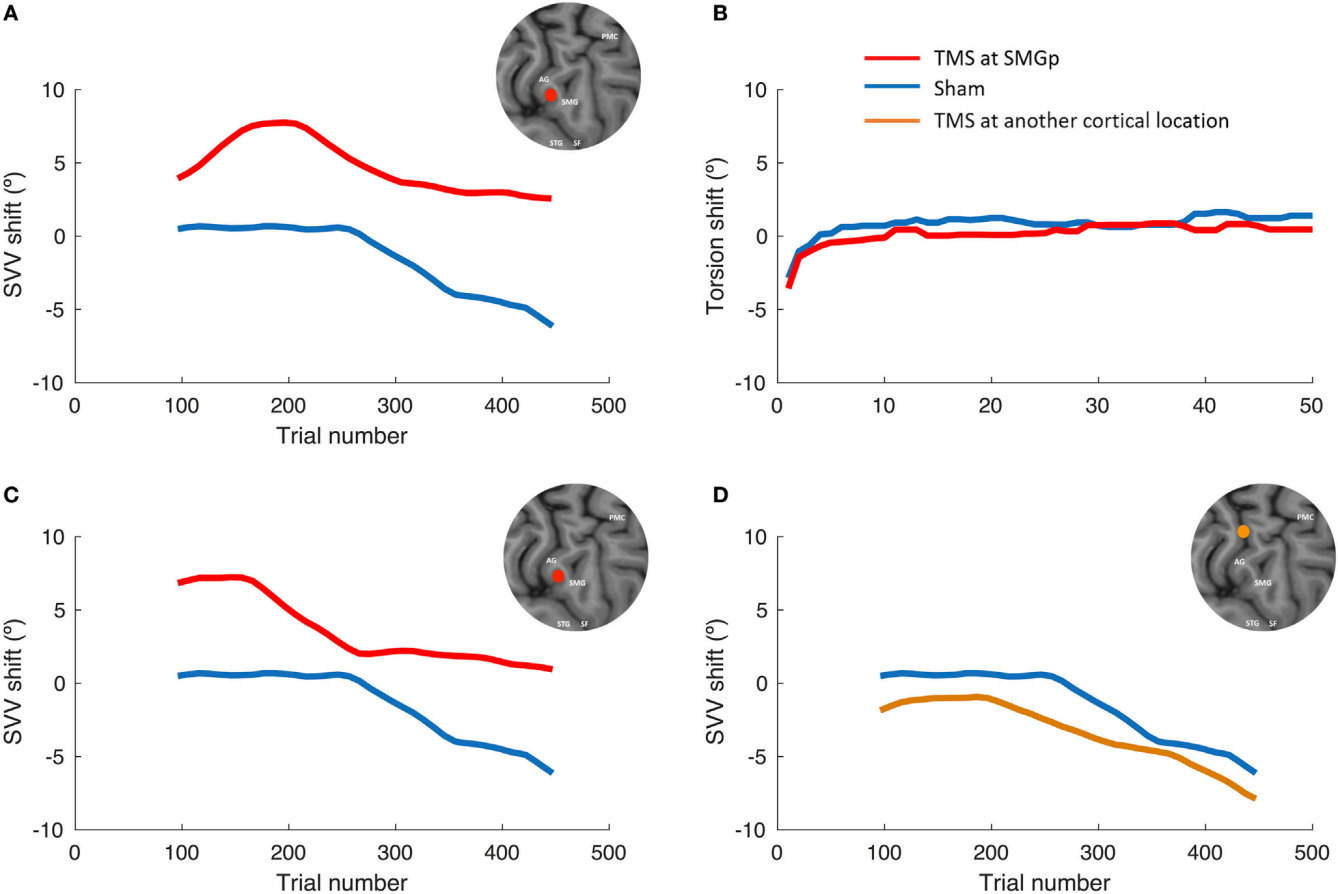
SVV and ocular torsion shifts following TMS and sham stimulation in a single participant. **(A)** SVV shift at the supramarginal gyrus (SMGp) (red) is shown along with the SVV shift from the sham session (blue). In both traces, there is a leftward drift over time (i.e., the head tilt direction), but the SVV shift from TMS is larger and it is in the opposite direction of the head tilt. **(B)** Ocular torsion shift is shown for SMGp (red) along with the sham session (blue). There is no significant difference between the sham and TMS sessions. **(C)** SVV shift from the localization session (first TMS session) at SMGp (red) is shown along with the sham session (blue). The SVV shift is comparable to the second TMS session at SMGp **(A)**, which shows a reproducible TMS effect at SMGp. **(D)** SVV shift at another cortical location (orange) is shown along the sham session (blue). Here, both traces also drift over time toward the left side (i.e., the head tilt direction); however, the SVV shift from TMS is smaller and it is in the same direction as the head tilt.

Figure 3 shows the SVV and ocular torsion shifts following TMS and sham stimulation in one participant. The SVV shift following TMS at SMGp is in the opposite direction of the head tilt, and it is larger compared to the sham stimulation. The SVV shift is also comparable to the first TMS session at SMGp, which was done to verify its cortical location. The ocular torsion, however, is not different between the TMS and sham sessions. As opposed to SMGp, the SVV shift following TMS at another cortical location is smaller and it is in the same direction of the head tilt.

**Figure 4:**
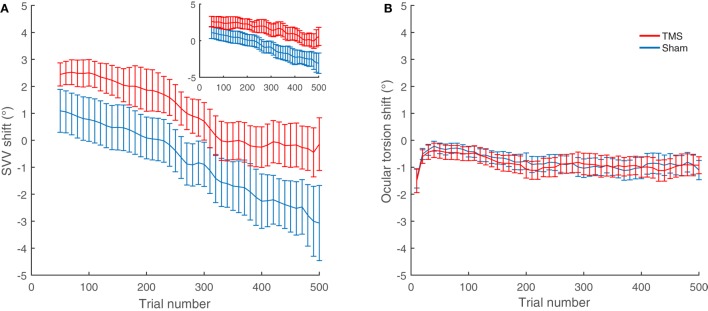
SVV and ocular torsion shifts following TMS and sham stimulation in all participants: **(A)** The average SVV shift from TMS at the supramarginal gyrus (SMGp) (red) is in the opposite direction of the head tilt and larger compared to the average SVV shift from the sham stimulation (blue). The small inset shows the similar result from the first TMS session at SMGp. **(B)** The average ocular torsion shift from TMS at SMGp (red) is not significantly different from the average ocular torsion shift from the sham stimulation (blue). Error bars represent SEM.

The average shifts in SVV and ocular torsion for all participants are also shown in Figure 4. The average SVV shift following TMS (1.0°) was significantly different from the average SVV shift following the sham stimulation (−0.8°) (*p* = 0.04). The positive SVV shift following TMS indicate that the shift was in the opposite direction of the head tilt (i.e., a rightward shift), and the negative SVV shift following the sham stimulation indicates that the shift was in the same direction as the head tilt (i.e., a leftward shift). Overall, the average SVV shift following TMS at SMGp was comparable to the first TMS session (SVV shift, first TMS session 1.6°; second TMS session 1.0°; *p* = 0.5), which shows that the SVV shift was reproducible at SMGp. The average shift in ocular torsion following TMS (−0.8°) was not different from the sham stimulation (−0.9°) (*p* = 0.8). Thus, the SVV shift induced by TMS was not accompanied by changes in the ocular torsion. As described previously, SVV values may drift over time during a static head tilt, usually toward the direction of the head tilt (10). Accordingly, here there was also an average SVV drift of −3.9° (i.e., leftward) in the TMS sessions and an average SVV drift of −4.8° in the sham sessions among participants. The slopes of these drifts were not different (*p* = 0.4). Therefore, although TMS resulted in an SVV shift in the opposite direction of the head tilt, there was no significant change in the SVV drift over time (Figure 4A).

**Figure 5:**
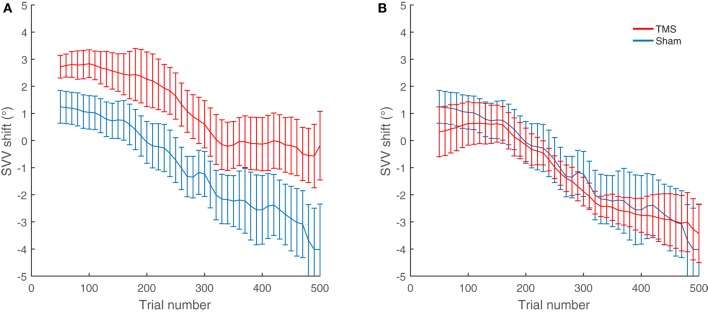
Average SVV shift following TMS and sham stimulation in nine participants who had TMS at different cortical locations. **(A)** The average SVV shift from TMS at SMGp is in the opposite direction of the head tilt and larger than the average SVV shift from the sham stimulation (*p* = 0.01). **(B)** The average SVV shift from TMS at other cortical locations is not different from the average SVV shift from the sham stimulation (*p* = 0.77). Error bars represent SEM.

Overall, nine participants also had TMS at other cortical locations near SMGp (Figure 2). The average SVV shift following TMS at these nearby cortical locations (−1.3°) was not different from the sham stimulation (−1.1°) (*p* = 0.77). We also compared the effect of TMS at SMGp with nearby cortical locations and the SVV shift following TMS at SMGp (1.1°) was significantly different from the SVV shift following TMS at nearby cortical locations (−1.3°) (*p* = 0.01) (Figure 5).

We also tested the possibility of TMS affecting SVV precision, which was calculated as the slope of the psychometric fits to the SVV responses. A change in precision would indicate that our results could be due to the effect of fatigue and not TMS *per se*. However, there was no difference between the SVV precisions following TMS at SMGp (0.3°) and the sham stimulation (0.36°) (*p* = 0.2). The trial reaction times were also compared and there was no significant difference between the TMS at SMGp (0.008 s) and the sham stimulation (−0.029 s) (*p* = 0.2). In both cases, the values compared were the shift in precision and reaction time, which were derived similar to the approach used for comparing SVV and torsion values.

## Discussion

Our results show that the inhibitory effect of TMS at SMGp (within TPJ) alters perception of upright without changing the torsional position of the eyes. This finding excludes a direct cortical influence on torsional eye position as a cause for the altered perception of upright. The SVV shift reported here is comparable with the similar effect of TMS at SMGp reported previously by our group (12). In line with this finding, patients with cortical lesions involving TPJ show SVV deviations without a change in the torsional eye position (21). In addition, the difference between the average SVV shift from TMS and sham stimulation in our results (1.83°) is compatible with the SVV deviations reported in patients with cortical lesions (1, 2, 21–23). These patients, however, might have had adaptive changes due to the chronic course of their deficits, and thus showed SVV deviations smaller than those seen in acute cortical lesions.

The temporoparietal junction is a cortical hub for various aspects of spatial perception including visuospatial attention, heading perception, visual gravitational motion, sense of embodiment, self-localization, and egocentricity (5, 6, 24–35). Accordingly, lesions in this cortical area have also been linked with out-of-body experience and room tilt illusion (31, 36–40). With respect to upright perception, TPJ involvement is especially evident in patients with neglect syndrome. These patients are unable to attend to sensory stimuli in their contralesional hemispace and also show significant contraversive deviations of their upright perception (1, 41–48). These lines of evidence suggest that same cortical networks contribute to perception of body orientation, visuospatial awareness, and upright orientation. From such perspective, the multisensory processing at TPJ would be crucial for the construction of reference frames in order to make extrapersonal spatial transformations. Our results are also in keeping with this view and show that SMGp is involved in processing sensory inputs underlying upright perception, without directly affecting the ocular torsion. Such sensory processing requires information about the head position in space, torsional eye position in the orbit (i.e., proprioceptive or efference copy signals), and visual inputs from the retina (7, 10). The brain must be able to incorporate these various sources of sensory information through a neural integration process, so that it can maintain a common reference frame for upright orientation. Therefore, the cortical effect of TMS at SMGp could be related to changes in individual sensory signals or computation of a common multisensory reference frame for perception of upright.

In this study, we used cTBS protocol for magnetic stimulation, which has an inhibitory cortical effect lasting for minutes (15). Because of this prolonged TMS effect, which may arise at different time points in individual subjects, we used long recording paradigms that could track temporal changes in SVV and ocular torsion. During a prolonged static head tilt, SVV responses usually change in the direction of the head tilt. This SVV change, although may have a variable time course, its overall trend can be calculated as the slope of a linear fit to the SVV responses (i.e., the SVV drift). During head tilt, the SVV drift does not correlate with the changes in torsional eye position and likely it is caused by the adaptive changes in sensory signals that encode head position (10, 49). As mentioned earlier, our participants showed similar SVV drifts—changing gradually in the same direction as the head tilt—and while TMS consistently induced an SVV shift in the *opposite* direction of the head tilt, it did not affect the slope of the SVV drift (Figures 4 and 5). This pattern suggests that the cortical effect of TMS at SMGp did not alter the adaptive changes in SVV during head tilt.

Here, we used an open-loop SVV paradigm that could track temporal changes in upright perception, without providing any feedback or constraining responses within a fixed range of probing angles. We found no change in the SVV precision or trial reaction times following TMS, which shows the shift in upright perception was mainly due to the altered accuracy and not variability in SVV responses or fatigue. The cortical locations in our participants were within the expected depth of the magnetic field generated by TMS coil (17, 18). Although TMS at other cortical locations showed no significant SVV shift, this approach does not allow us to exclusively link SMGp to perception of upright. It is also possible that TMS inhibited other nearby areas or neural networks, however, at the moment we cannot establish such links with perception of upright.

In conclusion, our results show that the inhibitory effect of TMS at SMGp, a cortical area within TPJ, altered upright perception without commensurate changes in ocular torsion. This finding has important implications for understanding the functional role of this cortical area in orientation constancy. Changes in ocular torsion can indeed affect perception of upright as it occurs at subcortical levels. The dissociation between upright perception and ocular torsion at SMGp suggests this cortical area, without directly affecting torsional eye position, is primarily involved in sensory processing for spatial orientation (e.g., the efference copy signals for torsional eye position). Future studies will have to investigate the neural mechanisms at TPJ that contribute to sensory processing for upright perception and spatial orientation.

## Ethics Statement

This study was carried out in accordance with the recommendations of the Johns Hopkins Institutional Review Board (IRB) with written informed consent from all subjects. All subjects gave written informed consent in accordance with the Declaration of Helsinki. The protocol was approved by the Johns Hopkins Institutional Review Board (IRB).

## Author Contributions

All authors have contributed in designing and performing the experiments and preparing the manuscript.

## Conflict of Interest Statement

The authors declare that the research was conducted in the absence of any commercial or financial relationships that could be construed as a potential conflict of interest.

## References

[1] Karnath H-O Dieterich M Spatial neglect – a vestibular disorder? Brain (2006) 129:293–305. 10.1093/brain/awh698 16371409

[2] Barra J Marquer A Joassin R Reymond C Metge L Chauvineau V Humans use internal models to construct and update a sense of verticality. Brain (2010) 133:3552–63. 10.1093/brain/awq311 21097492

[3] Lopez C Blanke O. The thalamocortical vestibular system in animals and humans. Brain Res Rev (2011) 67:119–46. 10.1016/j.brainresrev.2010.12.002 21223979

[4] Lopez C Blanke O Mast FW. The human vestibular cortex revealed by coordinate-based activation likelihood estimation meta-analysis. Neuroscience (2012) 212:159–79. 10.1016/j.neuroscience.2012.03.028 22516007

[5] Hansen KA Chu C Dickinson A Pye B Weller JP Ungerleider LG. Spatial selectivity in the temporoparietal junction, inferior frontal sulcus, and inferior parietal lobule. J Vis (2015) 15:15. 10.1167/15.13.15 26382006 PMC4578575

[6] Kaski D Quadir S Nigmatullina Y Malhotra PA Bronstein AM Seemungal BM. Temporoparietal encoding of space and time during vestibular-guided orientation. Brain (2016) 139:392–403. 10.1093/brain/awv370 26719385 PMC4805090

[7] De Vrijer MD Medendorp WP Gisbergen JAMV. Accuracy-precision trade-off in visual orientation constancy. J Vis (2009) 9:9. 10.1167/9.2.9 19271919

[8] Tarnutzer AA Fernando DP Kheradmand A Lasker AG Zee DS. Temporal constancy of perceived direction of gravity assessed by visual line adjustments. J Vestib Res (2012) 22:41–54. 10.3233/VES-2011-0436 22699152 PMC4066463

[9] Kheradmand A Gonzalez G Otero-Millan J Lasker A. Visual perception of upright: head tilt, visual errors and viewing eye. J Vestib Res (2016) 25:201–9. 10.3233/VES-160565 26890421 PMC4890624

[10] Otero-Millan J Kheradmand A. Upright perception and ocular torsion change independently during head tilt. Front Hum Neurosci (2016) 10:573. 10.3389/fnhum.2016.00573 27909402 PMC5112230

[11] Brandt T Dieterich M. Vestibular syndromes in the roll plane: topographic diagnosis from brainstem to cortex. Ann Neurol (1994) 36:337–47. 10.1002/ana.410360304 8080241

[12] Kheradmand A Lasker A Zee DS. Transcranial magnetic stimulation (TMS) of the supramarginal gyrus: a window to perception of upright. Cereb Cortex (2015) 25:765–71. 10.1093/cercor/bht267 24084127 PMC4318535

[13] Rossi S Hallett M Rossini PM Pascual-Leone A Safety, ethical considerations, and application guidelines for the use of transcranial magnetic stimulation in clinical practice and research. Neurophysiol Clin (2009) 120:2008–39. 10.1016/j.clinph.2009.08.016 PMC3260536 19833552

[14] Otero-Millan J Treviño C Winnick A Zee DS Carey JP Kheradmand A. The video ocular counter-roll (vOCR): a clinical test to detect loss of otolith-ocular function. Acta Otolaryngol (2017) 137:593–7. 10.1080/00016489.2016.1269364 28084887 PMC5502765

[15] Huang Y-Z Edwards MJ Rounis E Bhatia KP Rothwell JC. Theta burst stimulation of the human motor cortex. Neuron (2005) 45:201–6. 10.1016/j.neuron.2004.12.033 15664172

[16] Otero-Millan J Roberts DC Lasker A Zee DS Kheradmand A. Knowing what the brain is seeing in three dimensions: a novel, noninvasive, sensitive, accurate, and low-noise technique for measuring ocular torsion. J Vis (2015) 15:11. 10.1167/15.14.11 26587699 PMC4633118

[17] Brasil-Neto JP Cohen LG Panizza M Nilsson J Roth BJ Hallett M. Optimal focal transcranial magnetic activation of the human motor cortex: effects of coil orientation, shape of the induced current pulse, and stimulus intensity. J Clin Neurophysiol (1992) 9:132–6. 10.1097/00004691-199201000-00014 1552001

[18] Rudiak D Marg E. Finding the depth of magnetic brain stimulation: a re-evaluation. Electroencephalogr Clin Neurophysiol (1994) 93:358–71. 10.1016/0168-5597(94)90124-4 7525244

[19] Robertson EM Théoret H Pascual-Leone A. Studies in cognition: the problems solved and created by transcranial magnetic stimulation. J Cogn Neurosci (2003) 15:948–60. 10.1162/089892903770007344 14614806

[20] Vesia M Prime SL Yan X Sergio LE Crawford JD. Specificity of human parietal saccade and reach regions during transcranial magnetic stimulation. J Neurosci (2010) 30:13053–65. 10.1523/JNEUROSCI.1644-10.2010 20881123 PMC6633525

[21] Brandt T Dieterich M Danek A. Vestibular cortex lesions affect the perception of verticality. Ann Neurol (1994) 35:403–12. 10.1002/ana.410350406 8154866

[22] Yelnik AP Lebreton FO Bonan IV Colle FMC Meurin FA Guichard JP Perception of verticality after recent cerebral hemispheric stroke. Stroke (2002) 33:2247–53. 10.1161/01.STR.0000027212.26686.48 12215595

[23] Baier B Suchan J Karnath H-O Dieterich M. Neural correlates of disturbed perception of verticality. Neurology (2012) 78:728–35. 10.1212/WNL.0b013e318248e544 22357719

[24] Blanke O Slater M Serino A. Behavioral, neural, and computational principles of bodily self-consciousness. Neuron (2015) 88:145–66. 10.1016/j.neuron.2015.09.029 26447578

[25] Bosco G Carrozzo M Lacquaniti F. Contributions of the human temporoparietal junction and MT/V5+ to the timing of interception revealed by transcranial magnetic stimulation. J Neurosci (2008) 28:12071–84. 10.1523/JNEUROSCI.2869-08.2008 19005072 PMC6671632

[26] Cazzato V Mian E Serino A Mele S Urgesi C Distinct contributions of extrastriate body area and temporoparietal junction in perceiving one’s own and others’ body. Cogn Affect Behav Neurosci (2015) 15:211–28. 10.3758/s13415-014-0312-9 25047105

[27] Donaldson PH Rinehart NJ Enticott PG. Noninvasive stimulation of the temporoparietal junction: a systematic review. Neurosci Biobehav Rev (2015) 55:547–72. 10.1016/j.neubiorev.2015.05.017 26073069

[28] Igelström KM Graziano MSA. The inferior parietal lobule and temporoparietal junction: a network perspective. Neuropsychologia (2017) 105:70–83. 10.1016/j.neuropsychologia.2017.01.001 28057458

[29] Indovina I Maffei V Bosco G Zago M Macaluso E Lacquaniti F. Representation of visual gravitational motion in the human vestibular cortex. Science (2005) 308:416–9. 10.1126/science.1107961 15831760

[30] Lacquaniti F Bosco G Indovina I La Scaleia B Maffei V Moscatelli A Visual gravitational motion and the vestibular system in humans. Front Integr Neurosci (2013) 7:101 10.3389/fnint.2013.00101 24421761 PMC3872780

[31] Lopez C Halje P Blanke O. Body ownership and embodiment: vestibular and multisensory mechanisms. Neurophysiol Clin (2008) 38:149–61. 10.1016/j.neucli.2007.12.006 18539248

[32] Roberts RE Ahmad H Arshad Q Patel M Dima D Leech R Functional neuroimaging of visuo-vestibular interaction. Brain Struct Funct (2017) 222(5):2329–43. 10.1007/s00429-016-1344-4 27942855 PMC5504268

[33] Saj A Cojan Y Musel B Honoré J Borel L Vuilleumier P. Functional neuro-anatomy of egocentric versus allocentric space representation. Neurophysiol Clin (2014) 44:33–40. 10.1016/j.neucli.2013.10.135 24502903

[34] Silani G Lamm C Ruff CC Singer T. Right supramarginal gyrus is crucial to overcome emotional egocentricity bias in social judgments. J Neurosci (2013) 33:15466–76. 10.1523/JNEUROSCI.1488-13.2013 24068815 PMC6618458

[35] Ventre-Dominey J. Vestibular function in the temporal and parietal cortex: distinct velocity and inertial processing pathways. Front Integr Neurosci (2014) 8:53. 10.3389/fnint.2014.00053 25071481 PMC4082317

[36] Blanke O Ortigue S Landis T Seeck M Neuropsychology: stimulating illusory own-body perceptions. Nature (2002) 419:269–70. 10.1038/419269a 12239558

[37] Blanke O Landis T Spinelli L Seeck M. Out-of-body experience and autoscopy of neurological origin. Brain (2004) 127:243–58. 10.1093/brain/awh040 14662516

[38] Brandt T Strupp M Dieterich M Towards a concept of disorders of “higher vestibular function”. Front Integr Neurosci (2014) 8:47 10.3389/fnint.2014.00047 24917796 PMC4041089

[39] De Ridder D Van Laere K Dupont P Menovsky T Van de Heyning P Visualizing out-of-body experience in the brain. N Engl J Med (2007) 357:1829–33. 10.1056/NEJMoa070010 17978291

[40] Ionta S Heydrich L Lenggenhager B Mouthon M Fornari E Chapuis D Multisensory mechanisms in temporo-parietal cortex support self-location and first-person perspective. Neuron (2011) 70:363–74. 10.1016/j.neuron.2011.03.009 21521620

[41] Kerkhoff G Zoelch C. Disorders of visuospatial orientation in the frontal plane in patients with visual neglect following right or left parietal lesions. Exp Brain Res (1998) 122:108–20. 10.1007/s002210050497 9772118

[42] Kerkhoff G. Multimodal spatial orientation deficits in left-sided visual neglect. Neuropsychologia (1999) 37:1387–405. 10.1016/S0028-3932(99)00031-7 10606013

[43] Gentaz E Badan M Luyat M Touil N. The manual haptic perception of orientations and the oblique effect in patients with left visuo-spatial neglect. Neuroreport (2002) 13:327–31. 10.1097/00001756-200203040-00016 11930132

[44] Saj A Honore J Bernati T Coello Y Rousseaux M. Subjective visual vertical in pitch and roll in right hemispheric stroke. Stroke (2005) 36:588–91. 10.1161/01.STR.0000155740.44599.48 15705939

[45] Funk J Finke K Müller HJ Utz KS Kerkhoff G. Visual context modulates the subjective vertical in neglect: evidence for an increased rod-and-frame-effect. Neuroscience (2011) 173:124–34. 10.1016/j.neuroscience.2010.10.067 21073929

[46] Utz KS Keller I Artinger F Stumpf O Funk J Kerkhoff G. Multimodal and multispatial deficits of verticality perception in hemispatial neglect. Neuroscience (2011) 188:68–79. 10.1016/j.neuroscience.2011.04.068 21596103

[47] Karnath H-O Rorden C. The anatomy of spatial neglect. Neuropsychologia (2012) 50:1010–7. 10.1016/j.neuropsychologia.2011.06.027 21756924 PMC3348466

[48] Braem B Honoré J Rousseaux M Saj A Coello Y. Integration of visual and haptic informations in the perception of the vertical in young and old healthy adults and right brain-damaged patients. Neurophysiol Clin (2014) 44:41–8. 10.1016/j.neucli.2013.10.137 24502904

[49] Tarnutzer AA Bertolini G Bockisch CJ Straumann D Marti S. Modulation of internal estimates of gravity during and after prolonged roll-tilts. PLoS One (2013) 8:e78079. 10.1371/journal.pone.0078079 24205099 PMC3815095

